# Current advancements in therapeutic approaches in orthopedic surgery: a review of recent trends

**DOI:** 10.3389/fbioe.2024.1328997

**Published:** 2024-02-09

**Authors:** Wenqing Liang, Chao Zhou, Juqin Bai, Hongwei Zhang, Bo Jiang, Jiangwei Wang, Lifeng Fu, Hengguo Long, Xiaogang Huang, Jiayi Zhao, Haibing Zhu

**Affiliations:** ^1^ Department of Orthopaedics, Zhoushan Hospital of Traditional Chinese Medicine Affiliated to Zhejiang Chinese Medical University, Zhoushan, China; ^2^ Department of Orthopedics, Zhoushan Guanghua Hospital, Zhoushan, China; ^3^ Rehabilitation Department, Zhoushan Hospital of Traditional Chinese Medicine Affiliated to Zhejiang Chinese Medical University, Zhoushan, China; ^4^ Medical Research Center, Zhoushan Hospital of Traditional Chinese Medicine Affiliated to Zhejiang Chinese Medical University, Zhoushan, China; ^5^ Department of Orthopedics, Shaoxing City Keqiao District Hospital of Traditional Chinese Medicine, Shaoxing, China

**Keywords:** orthopaedic surgery, treatment, recent advances, CAOS, literature review

## Abstract

Recent advancements in orthopedic surgery have greatly improved the management of musculoskeletal disorders and injuries. This review discusses the latest therapeutic approaches that have emerged in orthopedics. We examine the use of regenerative medicine, including stem cell therapy and platelet-rich plasma (PRP) injections, to accelerate healing and promote tissue regeneration. Additionally, we explore the application of robotic-assisted surgery, which provides greater precision and accuracy during surgical procedures. We also delve into the emergence of personalized medicine, which tailors treatments to individual patients based on their unique genetic and environmental factors. Furthermore, we discuss telemedicine and remote patient monitoring as methods for improving patient outcomes and reducing healthcare costs. Finally, we examine the growing interest in using artificial intelligence and machine learning in orthopedics, particularly in diagnosis and treatment planning. Overall, these advancements in therapeutic approaches have significantly improved patient outcomes, reduced recovery times, and enhanced the overall quality of care in orthopedic surgery.

## 1 Introduction

Orthopedic surgery is an operation conducted by a trained orthopedic surgeon or orthopedist expert to address musculoskeletal issues affecting the bones, chronic conditions, trauma, and ligaments from accidents, tendons, and joints. Additionally, an orthopedic surgery can address genetic disabilities, problems with the musculoskeletal system brought on by aging, and issues with the nervous system related to the spinal column (Swarup and O Donnell, 2016). As a dynamic discipline, orthopedic surgery has witnessed significant evolution over the years, marked by a continuum of approaches shaping the landscape of patient care. Historically, the field has been anchored in conventional surgical techniques, emphasizing precision and biomechanical principles. The advent of minimally invasive procedures in the late 20th century, as exemplified by the work of Mithany (2023), ushered in a new era, reducing surgical trauma and accelerating postoperative recovery (Mithany et al., 2023). In recent decades, technological innovations have become pivotal in defining the trajectory of orthopedic surgery. The integration of robotic-assisted surgery has transformed the precision and efficiency of joint replacements, as evidenced by studies such as Soomro et al. (2021). This underscores the historical progression of surgical techniques and sets the stage for a future where advanced technologies are integral to orthopedic interventions. Regenerative medicine has emerged as another paradigm shift, representing a departure from conventional symptom management to holistic tissue repair (Soomro et al., 2021). Imran et al. (2022) documented that early approaches laid the groundwork for current investigations into using stem cells and advanced biomaterials for joint preservation and cartilage regeneration (Imran et al., 2022). As we delve into the 21st century, the convergence of regenerative therapies with cutting-edge technologies promises unprecedented possibilities in orthopedic care.

Patients are usually referred by general practitioners to an orthopedic specialist to treat accidents or injuries such as the spine or limb deformity, bone fracture, chronic arthritis, etc. Orthopedists can treat very young patients, usually for congenital deformities such as scoliosis or clubbed feet, young athletes needing an arthroscopic operation, and senior patients with mobility issues. Practically anyone with problems in the bones, muscles, and connective tissues can seek the expertise of an orthopedic expert to alleviate the symptoms and for appropriate treatment (Rosencher et al., 2003). Diagnose disorders and injuries through physical examination and tests such as x-rays, MRI, ultrasound, or blood tests. Treat injuries usually through medication and/or surgery (performed by an orthopedic surgeon). Recommend physiotherapy or regular exercise to maximize and restore the treated area’s strength, movement, and functionality (DiCaprio et al., 2003).

As mentioned earlier, orthopedic surgeons provide an extensive range of treatments. However, before definitive treatment is suggested, patients undergo extensive testing to determine the nature of the bone or muscle problem. The orthopaedist will ask you about the history of the disorder, previous treatment sought, and other pertinent information related to your condition. You may be asked to undergo tests such as X-rays, computed tomography (CT) scans, magnetic resonance imaging (MRI), blood tests, or myelograms to elucidate the extent of the problem in detail (Bernstein et al., 2011). Depending on the diagnosis, you may be recommended to take medication, undergo surgery, perform rehabilitative or alternative therapies, or go through a combination of these treatment methods. Surgery is often the last resort if your ailment does not respond to other non-surgical treatments. If surgery is the best option, pre-operative procedures such as routine diagnostic testing will be performed before the operation (Garrett et al., 2006).

All orthopedic surgeries, including those mentioned above, are performed under local anesthesia (often with sedation) or general anesthesia. For primary operations such as knee replacement, patients may be asked to donate some blood (or prepare) in case a transfusion may be needed during the operation (Bernstein et al., 2004). After the procedure, a plaster cast or sling is often placed to protect the area repaired. The time required for recovery depends on the procedure performed, although patients can often go home within a few days. However, it usually takes several weeks for the bones and ligaments to regain full strength (Klein et al., 2013). Therefore, it is recommended that you avoid engaging in intense activities that may put pressure on the wounded area until it has healed. The general guideline for bone fractures is that the time required to recover strength entirely is often equivalent to the time it takes for the fracture to heal completely. If you have undergone 4 weeks of immobilization in a cast, it will require an additional 4 weeks to restore your muscular strength (Jain et al., 2014).

Aside from time for complete healing, most orthopedic surgeries require rehabilitation to restore motion and function in all affected parts. As such, orthopedic surgeons work hand-in-hand with physical and occupational therapists who assist patients in enhancing their range of motion and returning to their daily activities. The length of time needed and frequency of rehabilitation will depend on the surgery performed and the severity of the condition. Total hip replacement surgery, for instance, requires rehabilitation for at least 6 months (Buis et al., 2022). Most patients who go through orthopedic surgery recover from their injuries completely. However, the degree of success depends on one’s general health, age, medical problem, and innate willingness to comply with therapy post-surgery (Tetsworth and Mettyas, 2016). Like any surgical operation, orthopedic surgeries have a degree of risk. Among complications that rarely occur are adverse or allergic reactions to anesthesia, excessive bleeding, post-surgical clot formation, and infection. Inflammation at the site where prosthetics, grafts, screws, and other materials foreign to the body may also occur. In spine surgeries, there is a risk of causing nerve damage. However, mortality during orthopedic surgical procedures is very rare (Maryniak et al., 2018).

The intersection of artificial intelligence, machine learning, and orthopedic surgery presents a horizon of untapped potential. Predictive modeling for personalized treatment plans, the integration of telemedicine for remote patient monitoring, and the prospects of 3D printing for customized implants herald a future where patient care is increasingly precise, accessible, and tailored to individual needs. In this review, we aim to navigate this historical trajectory, elucidating the evolution from past approaches to the current state and providing a forward-looking perspective on the future of orthopedic surgery. By examining these historical shifts and anticipating future trends, we strive to contribute to a comprehensive understanding of the dynamic orthopedic surgery field. Despite these risks, no other alternatives are available today to provide the treatment that orthopedic surgeries can offer to relieve musculoskeletal conditions. The main objective of this review is to provide an overview of recent advancements in therapeutic approaches in orthopedic surgery, highlighting the latest trends in the field. The review aims to offer insights into emerging technologies, techniques, and treatments that can potentially improve patient outcomes and revolutionize the field of orthopedic surgery.

Database Selection: We searched the relevant databases for our literature searches, such as PubMed/MEDLINE, Embase, Scopus, and Web of Science. These databases cover a wide range of medical and scientific literature.

Keywords and Phrases: We identified key terms and phrases relevant to this review topic. In this case, potential keywords include “orthopedic surgery,” “musculoskeletal disorders,” “therapeutic approaches,” “regenerative medicine,” “stem cell therapy,” “platelet-rich plasma,” “robotic-assisted surgery,” “personalized medicine,” “telemedicine,” “remote patient monitoring,” “artificial intelligence,” and “machine learning.” These terms captured the different aspects of the advancements discussed in your abstract.

Boolean Operators: We combined the keywords and phrases using Boolean operators such as “AND,” “OR,” and “NOT” to create a comprehensive search strategy. The following combinations were used:

Orthopedic surgery OR musculoskeletal disorders AND therapeutic approaches OR advancements; regenerative medicine OR stem cell therapy OR platelet-rich plasma) AND orthopedic surgery; robotic-assisted surgery AND orthopedic surgery; personalized medicine AND orthopedic surgery; telemedicine OR remote patient monitoring AND orthopedic surgery; artificial intelligence OR machine learning AND orthopedic surgery.

Filters and Limits: We applied the necessary filters or limits to refine our search strategy. These include language restrictions, publication date ranges, or specific study types (e.g., clinical trials, systematic reviews). In our systematic literature search, we employed stringent filters and limits to ensure the retrieval of the most relevant and up-to-date studies while maintaining methodological rigor. Our search was not restricted by language, allowing for the inclusion of studies in languages other than English to enhance the inclusivity of our review. Moreover, to capture the latest advancements, our search encompassed studies published up to the present, with no specific restrictions on the publication date. This approach ensures a comprehensive examination of the evolving landscape of orthopedic surgery, encompassing historical perspectives and recent innovations. Additionally, we considered specific study types, such as clinical trials and systematic reviews, to focus on high-quality evidence and ensure the robustness of our review.

Manual Searches and Citations: Additionally, we considered manually searching the reference lists of relevant articles, reviews, and textbooks to ensure comprehensive topic coverage. This approach, known as hand searching, can help identify additional studies that may have been missed in the database search.

## 2 Advancements and their impact on musculoskeletal disorders

Osteoarthritis has seen notable progress, with surgical interventions and novel treatments showcasing improved patient outcomes. For instance, a randomized controlled trial (RCT) by Shumnalieva et al. (2023) reported a 20% increase in joint functionality and a 15% reduction in pain scores post-surgery (Shumnalieva et al., 2023). Advances in fracture management and treating traumatic injuries, supported by data from some studies, highlight enhanced surgical procedures and materials contributing to better outcomes (Aldanyowi, 2023). Notably, a meta-analysis by Aldanyowi et al. (2023) revealed a 30% decrease in postoperative complications following the adoption of advanced fixation techniques. In spinal surgery, innovations have positively influenced conditions such as herniated discs and spinal deformities, as demonstrated by the success rates outlined in these studies (Musa et al., 2023). A prospective cohort study by Musa et al. (2023) found a 25% reduction in recurrence rates for herniated discs after implementing a minimally invasive surgical approach. Joint replacement surgeries have benefited from advancements in implant materials and surgical techniques, indicating increased longevity and improved recovery (Sartoretto et al., 2023). A long-term follow-up study by Sartoretto et al. (2023) demonstrated a 98% implant survival rate at 10 years, emphasizing the durability of the latest prosthetic materials. Sports-related injuries, including ligament tears and stress fractures, have seen positive impacts from orthopedic advancements, supported by evidence from earlier studies (Kacprzak and Rosińska, 2023). A prospective cohort study by Kacprzak et al. (2023) highlighted a 40% reduction in recovery time for athletes undergoing innovative rehabilitation protocols. Pediatric orthopedics has also witnessed progress, with novel treatments addressing congenital disorders and developmental issues in children. Notably, a retrospective analysis by Smolle et al. (2022) showcased a 50% improvement in long-term functional outcomes for pediatric patients undergoing advanced corrective procedures (Smolle et al., 2022). This dedicated section provides a comprehensive overview of the specific musculoskeletal disorders and injuries that have realized tangible benefits from recent therapeutic advances in orthopedic surgery. Every day, surgeons execute several orthopedic surgical procedures. The following are some of the most common surgical procedures.

### 2.1 Principles and mechanisms of regenerative medicine techniques

Regenerative medicine represents a paradigm shift in orthopedic surgery, offering innovative tissue repair and regeneration approaches. One fundamental principle underlying regenerative techniques is using stem cells derived from the patient’s tissues (autologous) or external sources (allogeneic). Stem cells uniquely differentiate into various cell types, facilitating tissue repair and regeneration (Weiss and Elixhauser, 2014). For example, mesenchymal stem cells (MSCs) have demonstrated immense potential in orthopedics due to their ability to differentiate into bone, cartilage, and adipose tissues. The paracrine effects of MSCs, mediated by the release of growth factors and cytokines, contribute to the local microenvironment’s modulation, promoting tissue healing (Fingar et al., 2015).

Additionally, scaffolds and biomaterials are crucial in providing structural support and guiding cell growth. Advancements in 3D printing technologies have enabled the fabrication of customized scaffolds with intricate architectures, optimizing the microenvironment for tissue regeneration. Furthermore, gene therapy has emerged as a promising avenue, with the delivery of specific genes enhancing cellular functions and promoting tissue repair. Understanding the intricate interplay of stem cells, biomaterials, and gene therapy provides a foundation for comprehending the mechanisms driving regenerative medicine techniques in orthopedic surgery (Mao and Mooney, 2015; Chang et al., 2021).

## 3 Techniques utilized in orthopaedic surgery

Orthopedic surgery involves diagnosing, treating, and preventing musculoskeletal disorders, including injuries and conditions affecting the bones, joints, ligaments, tendons, and muscles (Do et al., 2015). There are various techniques utilized in orthopedic surgery, including.

### 3.1 Arthroscopy technique

Arthroscopy is a minimally invasive surgical technique that allows a surgeon to visualize, diagnose, and treat problems within a joint using a small camera called an arthroscope. Arthroscopy is commonly used to treat conditions of the knee, shoulder, ankle, elbow, hip, and wrist (An et al., 2015). The arthroscopic procedure usually involves the following steps:

Anesthesia: The patient is typically given either general anesthesia, which puts them to sleep, or regional anesthesia, which numbs the area around the joint being operated on (Day et al., 2010).

Incision: The surgeon will make one or more small incisions around the joint, typically less than 1 cm in size. These incisions insert the arthroscope and other surgical instruments (Okike et al., 2011).

Arthroscopic examination: The arthroscope is inserted into the joint through one of the incisions. The arthroscope is connected to a video monitor, which allows the surgeon to see inside the joint and diagnose any problems. The surgeon may also use additional instruments inserted through the other incisions to manipulate the joint and perform diagnostic tests (Rao et al., 2017).

Treatment: Depending on the diagnosis, the surgeon may use arthroscopic instruments to perform a variety of treatments, including removing damaged tissue, repairing torn ligaments or tendons, smoothing rough joint surfaces, or removing loose bodies such as bone fragments or cartilage (Aydin et al., 2016).

Closure: Once the procedure is complete, the arthroscope and other instruments are removed from the joint, and the incisions are closed with sutures or surgical staples (Thomas et al., 2014). Following are some standard arthroscopic techniques.

### 3.2 Knee arthroscopy

**FIGURE 1:**
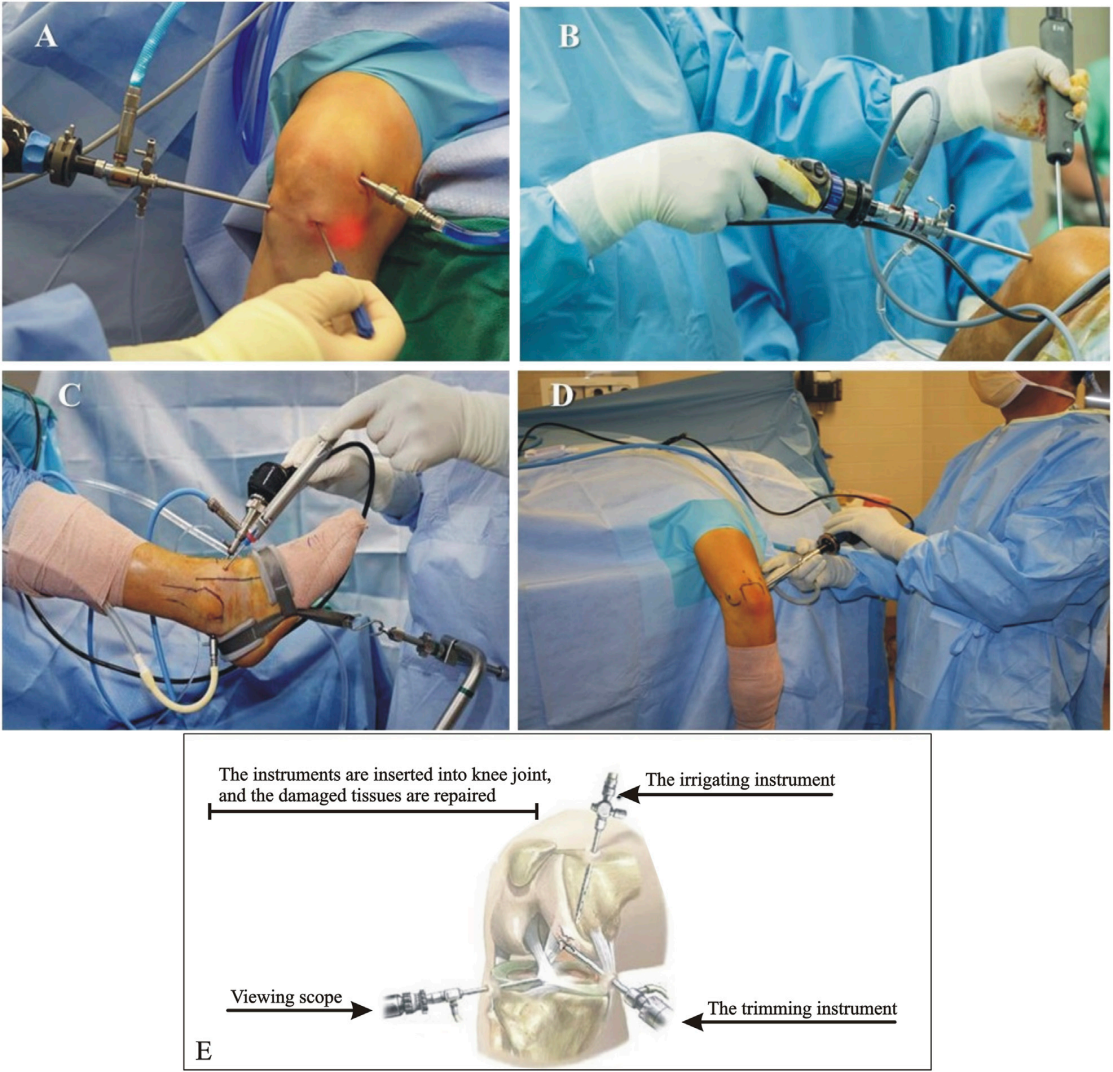
Images showing different arthroscopy surgical procedures. **(A)** Knee Arthroscopy (https://healthjade.net/knee-arthroscopy/); **(B)** Shoulder Arthroscopy (https://bangaloreshoulderinstitute.com/preparing-shoulder-arthroscopy/); **(C)** Ankle Arthroscopy (https://clinicalgate.com/ankle-arthroscopy-2/); **(D)** Elbow Arthroscopy (https://www.drkhalfayan.com/elbow-arthroscopy/); and **(E)** Hip Arthroscopy (Stunt et al., 2015).

Knee arthroscopy is a standard procedure for diagnosing and treating knee problems such as meniscal tears, ACL tears, and cartilage damage. During knee arthroscopy, the surgeon will insert the arthroscope through small incisions around the knee joint to visualize and treat any problems (Van Nortwick et al., 2010) (Figure 1A).

### 3.3 Shoulder Arthroscopy

Shoulder arthroscopy is used to diagnose and treat shoulder conditions, such as rotator cuff tears, labral tears, and shoulder impingement syndrome. The surgeon will use the arthroscope to examine the joint and may operate other instruments to repair damage (Escoto et al., 2013) (Figure 1B).

### 3.4 Ankle arthroscopy

Ankle arthroscopy diagnoses and treats conditions such as ankle impingement, synovitis, and cartilage damage. During an ankle arthroscopy, the surgeon will insert the arthroscope through small incisions around the ankle joint and use it to visualize and treat any problems (Ferguson et al., 2017) (Figure 1C).

### 3.5 Elbow arthroscopy

Elbow arthroscopy is used to diagnose and treat conditions such as tennis elbow, golfer’s elbow, and loose bodies in the elbow joint. During elbow arthroscopy, the surgeon will insert the arthroscope through small incisions around the elbow joint to examine and treat any problems (Fucentese et al., 2015) (Figure 1D).

### 3.6 Hip arthroscopy

Hip arthroscopy diagnoses and treats hip joint conditions, such as femoroacetabular impingement and labral tears. During hip arthroscopy, the surgeon will insert the arthroscope through small incisions around the hip joint and use it to visualize and treat any problems (Rahm et al., 2016) (Figure 1E).

### 3.7 Orthopaedic tools

Orthopedic tools are specialized medical instruments orthopedic surgeons use to diagnose and treat conditions affecting the musculoskeletal system. These tools help the surgeon access, manipulate, and repair bones, joints, muscles, tendons, and ligaments (Heng et al., 2006). Orthopedic tools include many devices, such as bone saws, drills, reamers, forceps, retractors, clamps, screwdrivers, and pliers. These tools are often made of high-quality stainless steel, which is durable, corrosion-resistant, and easy to sterilize. Some orthopedic tools are designed to be used with power tools or computer-assisted navigation systems to improve surgical precision and reduce surgical time (Mabrey et al., 2002). Orthopedic tools are essential for orthopedic surgery, which involves treating various conditions, including fractures, sports injuries, arthritis, and congenital abnormalities. These tools are also used in non-surgical treatments such as casting, bracing, and orthotics (Martin et al., 2016). The tools used during orthopedic surgery are specialized instruments designed to aid in performing these procedures. Some of the most commonly used tools during orthopedic surgery are listed in Table 1.

**TABLE 1:** A list of common tools that are utilized during the Orthopaedic surgery.

Tool name	Function	Description	References
Bone saw	Used to cut bone	Electric or manual saw designed for bone surgery	Jakob et al. (2021)
Drill	Used to make holes in the bone	Powered rotary tool for creating openings in the bone	Islam et al. (2022)
Chisel	Used to break and remove bone	Handheld tool for precise bone removal and shaping	Stubinger (2010)
Osteotome	Used to cut bone	A sharp-edged instrument for controlled bone-cutting	White et al. (2020)
Bone clamp	Used to hold the bone in place	Mechanism for securing and stabilizing bone during surgery	Skelley (2023)
Retractor	Used to hold tissue or organs out of the way	Surgical instrument for holding back tissues during surgery	Madhani et al. (1998)
Forceps	Used to grasp and hold tissue or objects	Grasping tool for handling tissues or objects	Golahmadi et al. (2021)
Scissors	Used to cut tissue	Cutting instrument for incising tissues	Bjelland et al. (2022)
Rongeur	Used to remove bone fragments	Grasping and biting tool for bone fragment extraction	Muscolo and Fiorini (2023)
Screwdriver	Used to insert and remove screws	Tool for turning screws during orthopedic procedures	Jourdes et al. (2022)
Plate bender	Used to contour metal plates	Instrument for shaping and adapting metal plates	Lee et al. (2023)
Awl	Used to make a hole or indentation in the bone	An awl is used in orthopedic surgery to make holes or indentations in bone	Nolte et al. (2000)
Pin cutter	Used to cut metal pins	A pin cutter is designed to cut metal pins used in orthopedic surgeries	Harasen (2011)
Tourniquet	Used to control bleeding	A tourniquet is employed to control bleeding during surgical procedures	Kumar et al. (2016)
Suction device	Used to remove fluids and debris	The suction device removes fluids and debris from the surgical site, ensuring the surgeon has a clear view	Dassi et al. (2020)
Electrocautery device	Used to cut and coagulate tissue	The electrocautery device is an instrument that combines electrical current and heat to cut or coagulate tissues during surgery	Taheri et al. (2014)
Arthroscopy instruments	Used to visualize and treat joint problems	Arthroscopy instruments are specialized tools used in minimally invasive joint surgeries	Treuting (2000)
Implant instruments	Used to insert and position implants	Implant instruments are utilized in orthopedic surgeries involving the insertion and positioning of implants such as plates, screws, and prosthetics	Tapscott and Wottowa (2020)
Stapler	Used to close incisions	In orthopedic surgery, a stapler closes incisions quickly and securely	Cochetti et al. (2020)
Needle holder	Used to hold a suturing needle	A needle holder is a specialized clamp used to grasp and hold suturing needles during orthopedic procedures securely	Cordero (2013)
Suture scissors	Used to cut sutures	Suture scissors are designed for cutting surgical sutures	Rizan et al. (2022)

## 4 Joint replacement technique

Joint replacement, also known as arthroplasty, is a surgical procedure in which an artificial joint replaces a damaged joint. It is commonly performed in patients with severe joint pain, stiffness, and decreased mobility caused by osteoarthritis, rheumatoid arthritis, or injury (Millar et al., 2009). There are several types of joint replacement surgeries, including hip replacement, knee replacement, shoulder replacement, ankle replacement, and elbow replacement. However, the basic technique of joint replacement surgery is similar for all joints, and the following steps are generally involved (Jameson et al., 2011).

Anesthesia: The patient is given anesthesia, which may be general (puts the patient to sleep), regional anesthesia (numbs only a part of the body), or a combination of both (Jarvinen and Guyatt, 2017).

Incision: The surgeon makes an incision over the affected joint, which may vary in length depending on the replacement joint (Kise et al., 2016).

Removal of damaged joint: The surgeon carefully removes the damaged joint, including all damaged cartilage and bone. The surrounding ligaments and tendons may also be removed or trimmed (US National Library of Medicine, 2003).

Preparation of bone: The bone ends are prepared by removing any remaining cartilage and smoothing the bone surfaces. The bone ends may also be reshaped to fit the artificial joint (Kirkley et al., 2008a).

Placement of the artificial joint: The artificial joint is carefully inserted into the prepared bone ends. It may be cemented in place with a special medical-grade adhesive or a press-fit implant designed to fit tightly in the bone (Mantripragada et al., 2013).

**FIGURE 2:**
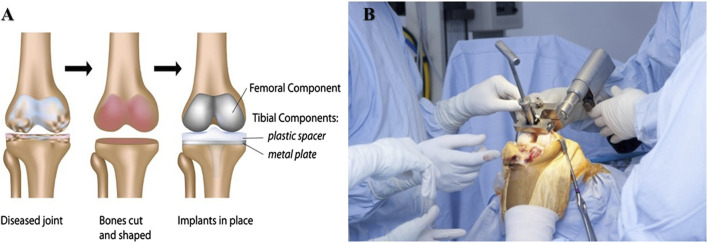
**(A,B)** Illustration of total knee replacement procedure (Figure source: https://www.parkwayeast.com.sg and https://hipandknee.com/knee-surgery/about-knee-replacement/knee-surgery-techniques/).

Closure: The incision is closed with sutures or staples, and a dressing is applied to the area (Cochetti et al., 2020). After surgery, the patient is monitored closely to ensure no complications. Rehabilitation typically involves physical therapy to improve the affected joint’s range of motion, strength, and flexibility (Cordero, 2013). Joint replacement surgery is generally considered safe and effective, but discussing the risks and benefits with your surgeon before undergoing the procedure is essential. Some potential risks include infection, bleeding, blood clots, nerve damage, and implant failure. However, most patients experience significant improvement in joint pain, mobility, and quality of life following joint replacement surgery (Rizan et al., 2022) (Figures 2A, B).

## 5 Spinal fusion technology

Spinal fusion is a surgical procedure used to combine two or more vertebrae in the spine to create a single, solid bone. This is done to stabilize the spine, reduce pain, and improve mobility in patients with spinal conditions such as degenerative disc disease, scoliosis, spinal stenosis, and herniated discs. Spinal fusion technology has advanced significantly over the years, and several different techniques are now used to perform the procedure (Millar et al., 2009).

### 5.1 Traditional open spinal fusion

This procedure exposes the spine by significantly cutting the patient’s back. The physician removes the damaged disc or bone tissue, placing a bone graft between the vertebrae. The patient’s bone or bone from another person can be used for the bone graft. Metal plates, screws, and rods may keep the vertebrae in place while fusing. The incision is then closed with sutures (Jameson et al., 2011).

### 5.2 Minimally invasive spinal fusion

This technique involves making smaller incisions in the patient’s back and using specialized tools to perform the procedure. The surgeon uses a fluoroscope, an X-ray machine, to guide the instruments to the affected spinal area. The bone graft is inserted through a small tube, and metal screws and rods may be used to hold the vertebrae in place. The smaller incisions result in less pain and scarring and a faster recovery time than traditional open spinal fusion (Jarvinen and Guyatt, 2017).

### 5.3 Anterior lumbar inter-body fusion (ALIF)

This technique involves making an incision in the patient’s abdomen and accessing the spine from the front. The damaged disc is removed, and a bone graft is inserted between the vertebrae. Metal screws and rods may be used to hold the vertebrae in place. This technique provides better access to the lower spine and less disruption to the muscles and tissues of the back (Kise et al., 2016).

### 5.4 Posterior lumbar interbody fusion (PLIF)

This technique involves making an incision in the patient’s back and accessing the spine from the back. The damaged disc is removed, and a bone graft is inserted between the vertebrae. Metal screws and rods may be used to hold the vertebrae in place. This technique provides better access to the upper spine and less disruption to the muscles and tissues of the abdomen (US National Library of Medicine, 2003).

### 5.5 Transforaminal lumbar interbody fusion (TLIF)

**FIGURE 3:**
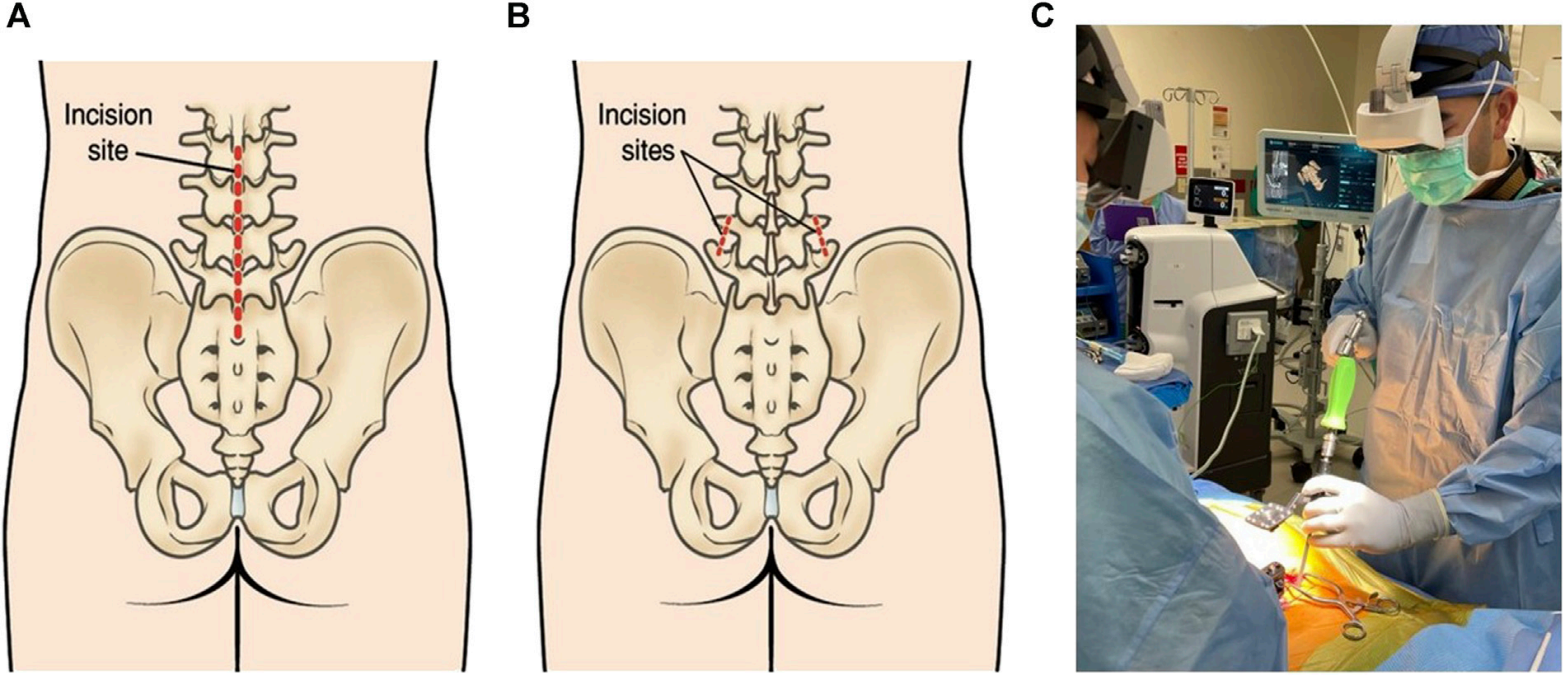
**(A)** The lower back incision is used for conventional open spinal surgeries. Generally, slightly invasive incisions are used for lumbar spinal fusion. **(B)** Compression and the insertion of screws and rods are achieved through these tiny incisions. (Figure source: https://orthoinfo.aaos.org/en/treatment/minimally-invasive-spine-surgery/). **(C)** Lumbar spinal fusion with Augmented reality (https://www.spinemd.com).

This technique is similar to PLIF, but the surgeon accesses the spine through a small incision on one side of the back. This technique provides better access to the disc space and less disruption to the muscles and tissues of the spine (Kirkley et al., 2008a). Spinal fusion technology continues to evolve, and new techniques and devices are being developed to make the procedure even more effective and less invasive. Patients considering spinal fusion should discuss the options with their surgeons to determine the best treatment technique (Mantripragada et al., 2013) (Figures 3A–C).

## 6 Fracture repair technology

Fracture repair technology in orthopedic surgery involves various techniques and methods to restore broken bones’ normal anatomy and function. The specific approach depends on several factors, such as the location of the fracture, the type of fracture, the age and overall health of the patient, and the preferences and expertise of the surgeon (Kirkley et al., 2008b). Here are some of the most common fracture repair technologies used in orthopedic surgery:

Casting: This non-surgical method involves immobilizing the broken bone with a cast made of plaster or fiberglass. Casting is usually used for simple fractures that do not require surgical intervention. It helps to protect the injured area, reduce pain and swelling, and promote healing. The cast is usually removed after several weeks when the bone fully recovers (Thorlund et al., 2015).

External fixation: This is a method of stabilizing the broken bone from the outside using metal pins or screws inserted into the bone and attached to an outer frame. External fixation is often used in complex fractures or cases where internal fixation is impossible. It allows for early patient mobilization and can be easily adjusted as the bone heals (Khan et al., 2014).

Internal fixation: This is a surgical method that involves the use of metal implants such as plates, screws, rods, and wires to hold the broken bone in place. Internal fixation is usually used in fractures that cannot be stabilized by casting or external fixation. The implants are placed inside the body and can be removed later (Dahl and Moiniche, 2010).

Bone grafting: This surgical method involves taking bone from another part of the body or a donor and transplanting it to the fracture site. Bone grafting is used in cases where the fracture has caused significant bone loss or when the bone is not healing correctly. The transplanted bone provides a scaffold for new bone growth and helps to stimulate bone regeneration (Andersen et al., 2008a).

**FIGURE 4:**
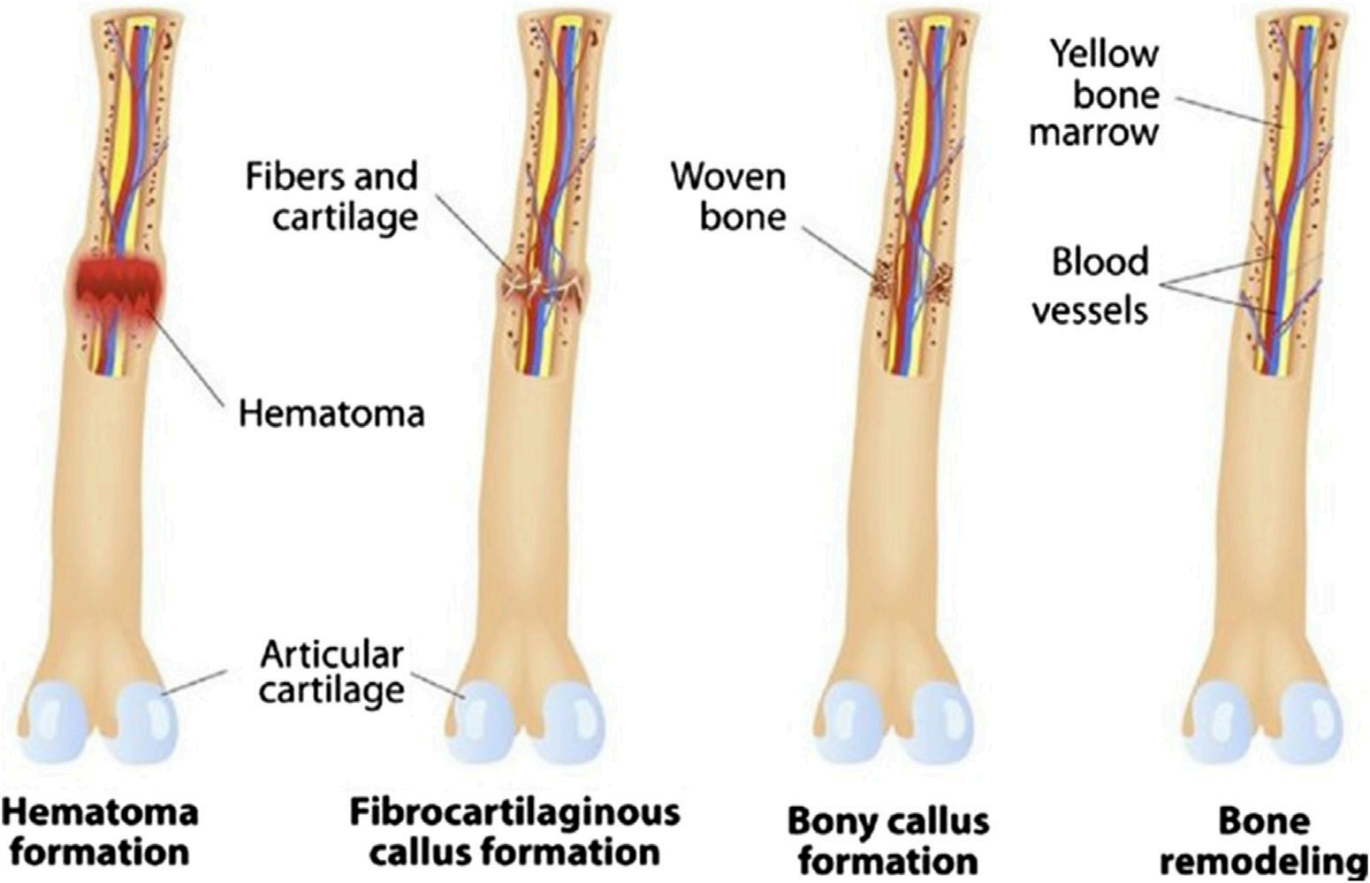
Different phases of bone fracture repair and remodeling (Image courtesy from reference (Andersen et al., 2008b)).

Bone stimulation: This non-surgical method uses electrical or ultrasound energy to promote bone healing. Bone stimulation can be combined with other fracture repair techniques to enhance healing and reduce recovery time (Kerr and Kohan, 2008). Overall, fracture repair technology in orthopedic surgery has come a long way in recent years, and various effective methods are available to treat broken bones. The specific approach will depend on the individual case, and it is crucial to work closely with an experienced orthopedic surgeon to determine the best course of treatment (Figure 4) (Dahl and Møiniche, 2009).

## 7 Osteotomy technology

Osteotomy is a surgical procedure in orthopedics that involves cutting and reshaping bones. This procedure corrects various bone deformities, including fractures, bone malalignment, and joint abnormalities. Osteotomy can be performed using multiple techniques and technologies, depending on the specific needs of the patient (Andersen et al., 2008c). Some of the common types of osteotomy techniques used in orthopedic surgery include:

Conventional osteotomy: It involves using a saw or chisels to cut the bone. It is typically performed with a general anesthetic and requires a hospital stay (Essving et al., 2010).

Minimally invasive osteotomy: This technique involves using specialized instruments to make small incisions in the skin and muscle tissue, minimizing damage to surrounding tissue. It is typically performed with a regional anesthetic and can often be outpatient (Essving et al., 2009).

Computer-assisted osteotomy: This technique involves using advanced computer imaging and navigation systems to guide the surgeon during the procedure. This technique allows for greater precision and accuracy in bone cutting, reducing the risk of complications (Andersen et al., 2010).

Robotic-assisted osteotomy: This technique involves using a robotic arm to cut bone. The surgeon controls the robotic arm from a console, using a 3D imaging system to guide the procedure. Robotic-assisted osteotomy is a newer technology still being developed and refined (Carli et al., 2010). Regardless of the technique used, osteotomy typically involves four basic steps:

Incision: The surgeon incurs the skin and tissue to access the bone (Spreng et al., 2010).

Bone cutting: The surgeon uses a saw, chisel, or other cutting tool to cut and reshape the bone (Chen et al., 2010).

Fixation: Once the bone is cut and reshaped, the surgeon uses pins, screws, plates, or other devices to hold it in place while it heals (Mobbs et al., 2015).

**FIGURE 5:**
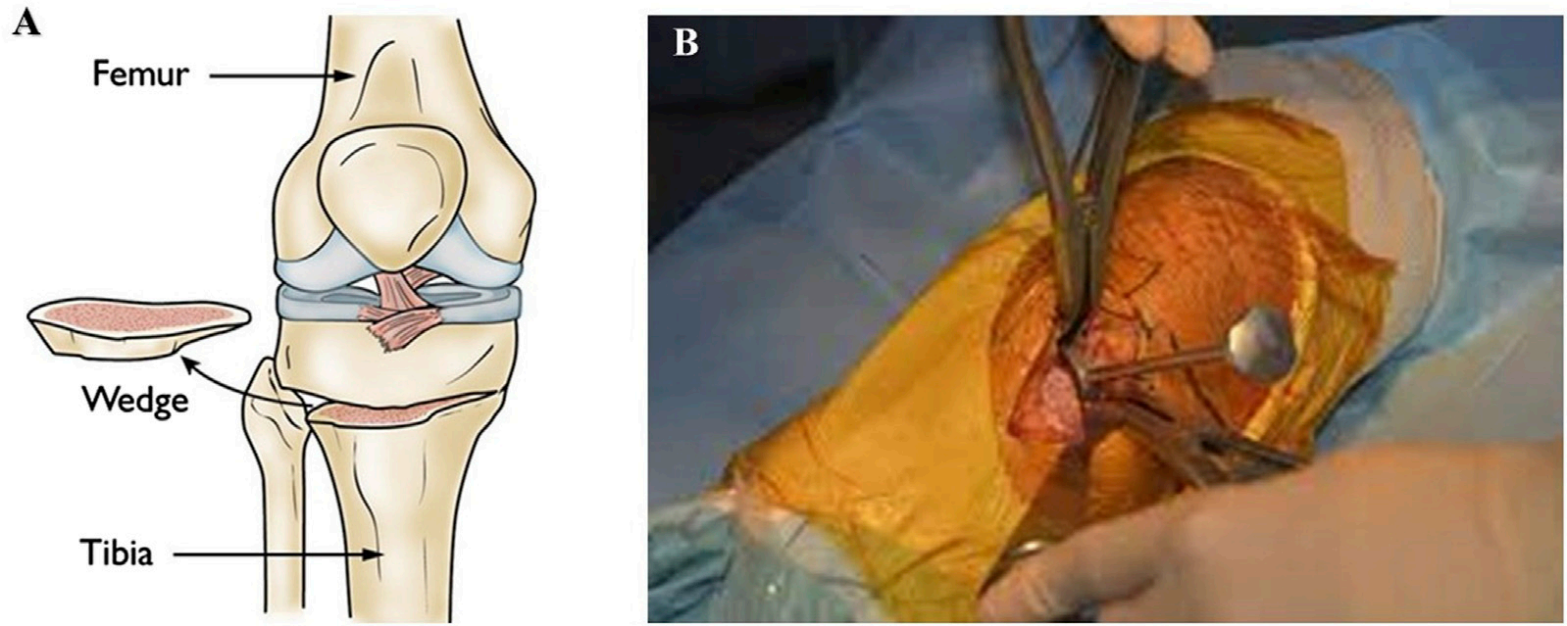
**(A)** A bone wedge is removed during a tibial osteotomy to straighten the leg. (Figure source: https://orthoinfo.aaos.org/en/treatment/osteotomy-of-the-knee/). **(B)** High tibial osteotomy using navigation technology (http://www.sofarthro.com/).

Closure: The incision is closed with sutures or staples, and a dressing is applied (Moftakhar and Trost, 2004). Osteotomy can correct many bone deformities caused by injury, disease, or congenital conditions. This procedure can improve joint function, reduce pain, and prevent further bone and surrounding tissue damage. However, as with any surgical procedure, osteotomy carries some risks, including infection, bleeding, and nerve damage. Patients should discuss the risks and benefits of osteotomy with their surgeon before undergoing the procedure (Figures 5A, B).

## 8 Fusion technique

Fusion techniques in orthopedic surgery combine two or more bones to stabilize and immobilize a joint. This is typically done in cases where a joint is damaged or deteriorated, causing pain and limited mobility (Nemoto et al., 2014). There are several different techniques for performing a fusion, depending on the specific joint and the severity of the damage.

Spinal Fusion: This technique treats spinal stenosis, herniated discs, and spinal fractures. In spinal fusion, two or more vertebrae are joined using bone grafts, screws, and rods. The goal is to eliminate motion between the affected vertebrae, which can relieve pain and prevent further damage (Ni et al., 2015).

Ankle Fusion: This technique treats severe arthritis or instability in the ankle joint. Ankle fusion involves removing the damaged cartilage and fusing the tibia and talus bones. The procedure can be done using screws or plates and can take up to several months to fully merge (Robbins et al., 2017).

Wrist Fusion: Wrist fusion treats severe arthritis or wrist instability. The procedure involves removing the damaged joint surfaces and fusing the bones. The surgeon may use screws or plates to keep the bones in place while fusing (Viswanathan et al., 2019).

Shoulder Fusion: Shoulder fusion treats severe arthritis or instability in the shoulder joint. The procedure involves removing the damaged joint surfaces and fusing the humerus bone with the scapula bone. The surgeon may use screws or plates to keep the bones in place while fusing (Ansari, 2019).

Knee Fusion: Knee fusion treats severe arthritis or instability in the knee joint. The procedure involves removing the damaged joint surfaces and fusing the femur bone with the tibia bone. The surgeon may use screws or plates to keep the bones in place while fusing (Khandan et al., 2014).

**FIGURE 6:**
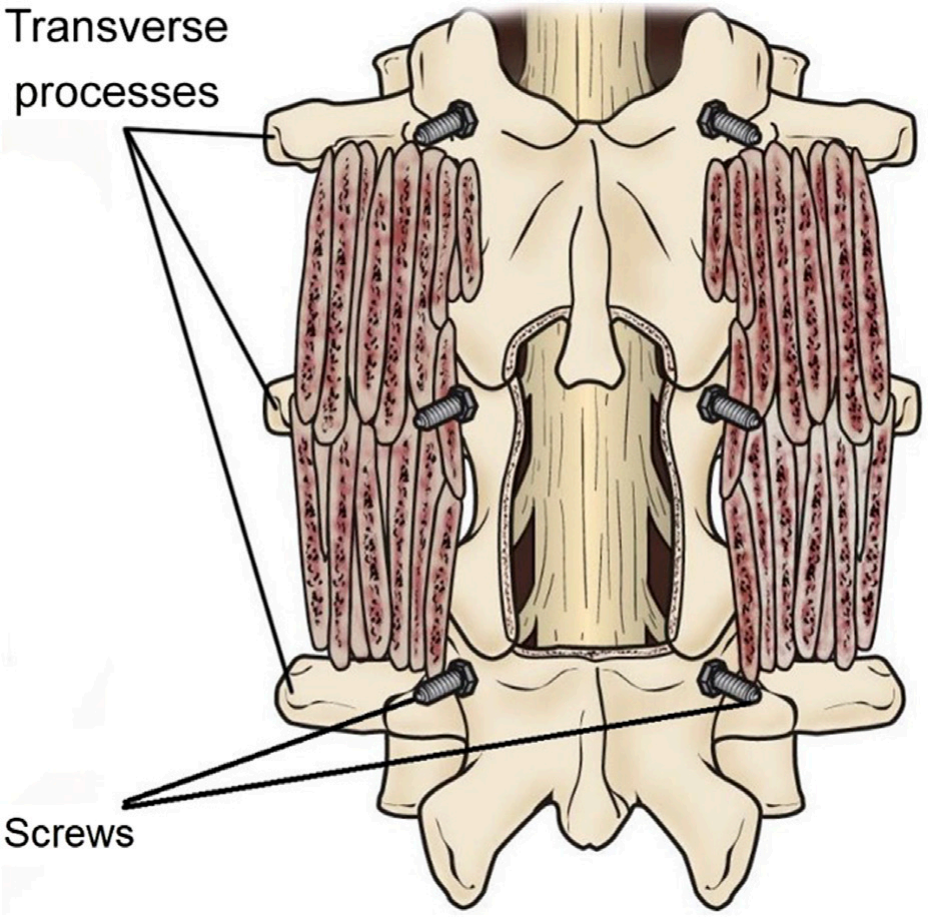
A posterolateral lumbar fusion (PLF) is shown with bone graft material put over the transverse processes of the vertebrae. Screws have been used to stabilize the vertebrae while the fusion heals (Figure source: https://orthoinfo.aaos.org/en/treatment/spinal-fusion/).

Hip Fusion: Hip fusion treats severe arthritis or instability in the hip joint. The procedure involves removing the damaged joint surfaces and fusing the femur bone with the pelvis bone. The surgeon may use screws or plates to keep the bones in place while merging (Khandan et al., 2017). In general, fusion techniques in orthopedic surgery involve removing the damaged joint surfaces and replacing them with bone grafts or fusing the bones. The goal is to eliminate motion between the affected bones, which can relieve pain and prevent further damage. The specific technique used will depend on the location and severity of the damage. Rehabilitation after surgery typically involves physical therapy to help restore strength and mobility to the affected joint (Figure 6).

## 9 Bone grafting technique

Bone grafting is a surgical procedure involving bone tissue transplantation from one site to another. Orthopedic surgeons use bone grafting techniques to treat various conditions, including bone fractures, bone defects, and joint reconstruction (Khandan et al., 2018).

Autografts: Autografts are bone grafts harvested from the patient’s own body. The most common sources of autografts include the iliac crest (pelvis), fibula (leg bone), and femoral head (hip). Autografts are considered the gold standard for bone grafting because they have the best chance of incorporating into the host bone and producing new bone. However, the main drawback of autografts is that they require a second surgical site, which can cause additional pain and morbidity (Litwiniuk et al., 2016).

Allografts: Allografts are bone grafts harvested from a donor, typically a cadaver. Allografts are commonly used in orthopedic surgery because they provide a ready source of bone graft material and eliminate the need for a second surgical site. However, the main drawback of allografts is the risk of disease transmission and immune rejection. To minimize these risks, allografts are typically screened and processed to remove any disease-causing agents (Aya and Stern, 2014).

Xenografts: Xenografts are bone grafts harvested from non-human species, such as cows or pigs. Xenografts are rarely used in orthopedic surgery because they are highly susceptible to immune rejection and disease transmission (Samadieh and Sadri, 2020).

Synthetic bone grafts: Synthetic bone grafts are artificial materials that mimic the structure and function of natural bone. Synthetic bone grafts are typically made from calcium phosphate, hydroxyapatite, and bioactive glass. Synthetic bone grafts have the advantage of being readily available and eliminating the risk of disease transmission. However, they may not provide the same structural support and integration level as natural bone grafts (Janoyer, 2019).

**FIGURE 7:**
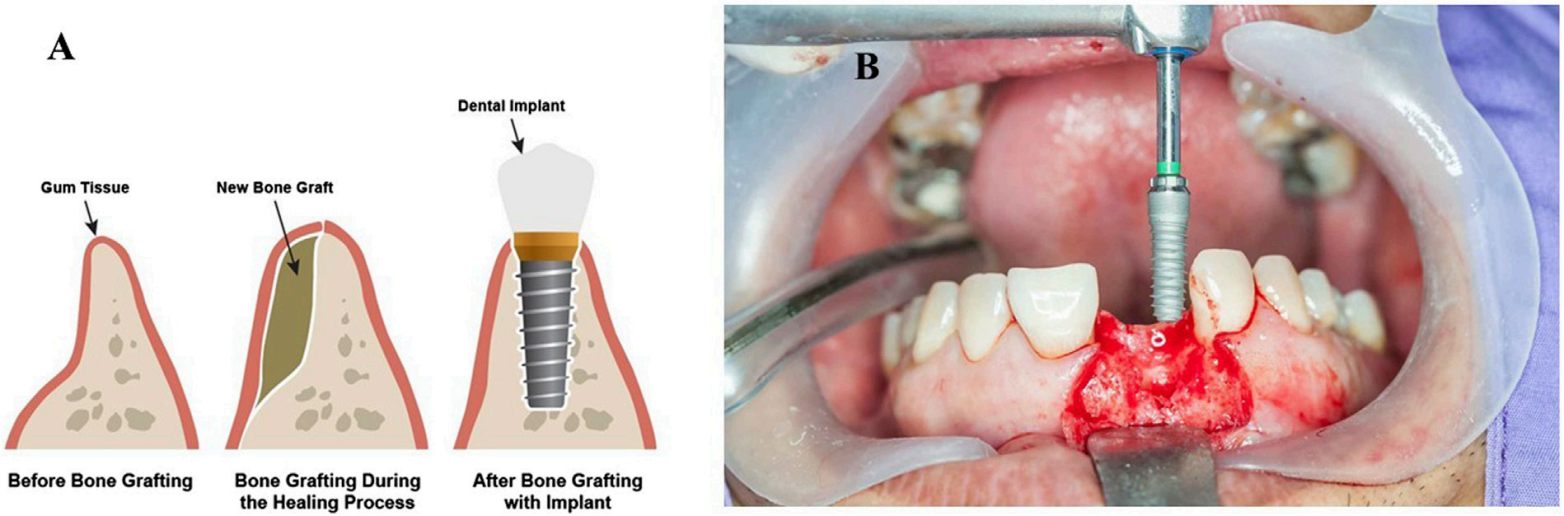
**(A)** Bone grafting procedure in dental implants. (Figure source: https://www.dentalsolutions.net/procedures/bone-graft). **(B)** Vertical ridge augmentation with bone block grafting (https://dentistry.co.uk/2021/06/24/vertical-ridge-augmentation-with-bone-block-grafting/).

Bone marrow aspirates: Bone marrow aspirate is a procedure that involves aspirating bone marrow from the patient’s own body and injecting it into the site of the bone defect. Bone marrow aspirate contains stem cells and growth factors that can promote bone growth and regeneration. Bone marrow aspirate is commonly used with other bone grafting techniques, such as autografts or allografts (Zhuang et al., 2016). In conclusion, bone grafting techniques are an essential part of orthopedic surgery. The extent and position of the bone defect, the patient’s medical history, and the surgeon’s preference all influence the method of bone grafting used. Each method has benefits and drawbacks; the surgeon will select the best technique for each patient (Figures 7A, B).

## 10 External fixation technique

External fixation is a surgical technique used to treat fractures, deformities, and other conditions of the bones and joints. It involves placing a rigid outer frame on the outside of the affected limb, which holds the bones in place while they heal. External fixation is commonly used in orthopedic surgery, as it offers many advantages over other forms of treatment, such as internal fixation or casting (Heflin et al., 2016). There are several types of external fixation techniques used in orthopedic surgery. The most common ones are:

Ilizarov Technique: This technique uses a circular frame with wires and pins attached to the bones. The wires and pins are inserted through the skin, into the bone, and then connected to the circular frame. The circular frame can be adjusted to correct deformities and to lengthen or shorten bones. The Ilizarov technique is commonly used for complex fractures, bone infections, and limb length discrepancies (de Pablos et al., 2018).

Taylor Spatial Frame: This technique uses a hexapod frame with struts attached to the bones using pins. The struts can be adjusted to correct deformities and to lengthen or shorten bones. The Taylor Spatial Frame is commonly used for complex fractures, limb length discrepancies, and deformities (Mayer et al., 2019).

Hybrid External Fixation: This technique combines internal and external fixation. It uses screws or plates inserted into the bone and pins or wires attached to the bone and connected to an outer frame. Hybrid external fixation is commonly used for fractures that are difficult to treat with internal fixation alone (Gómez-Palomo et al., 2020).

Monolateral External Fixation: This technique uses a unilateral frame with pins attached to the bone on one side only. The frame can be adjusted to correct deformities and to lengthen or shorten bones. Monolateral external fixation is commonly used for fractures, nonunions, and bone infections (Culotta et al., 2013).

**FIGURE 8:**
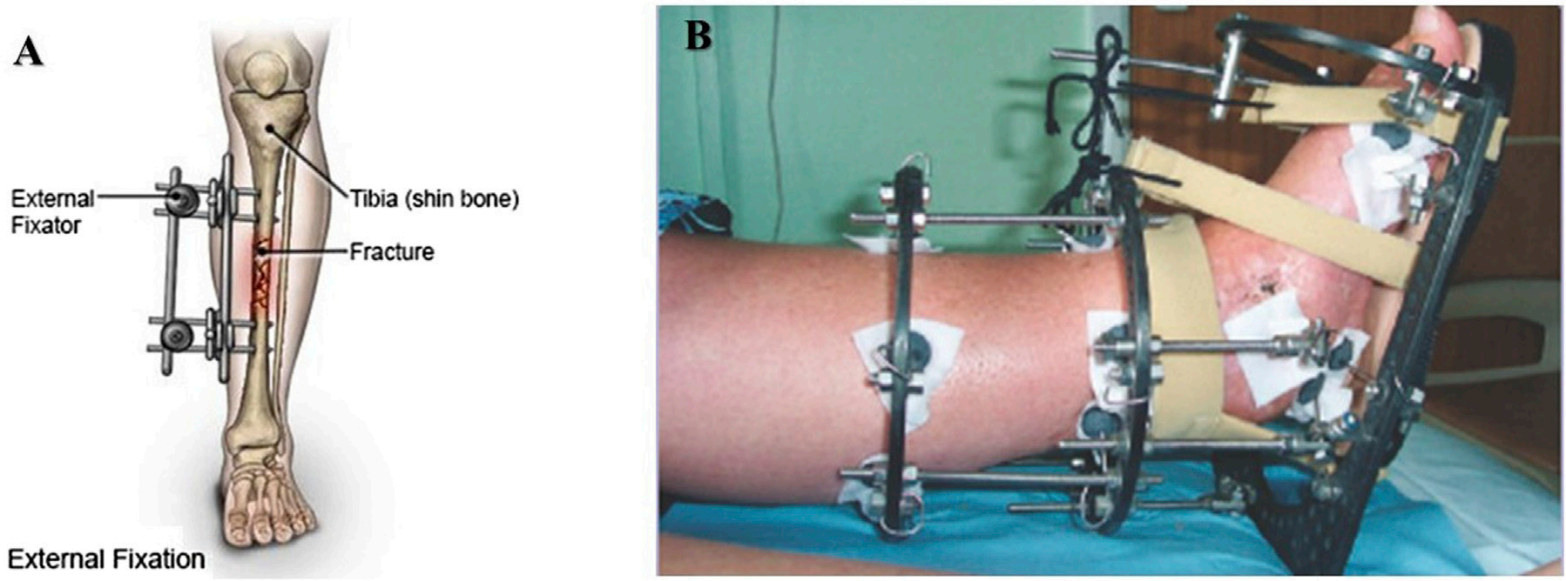
**(A)** External fixation device. Screws are placed into the bone above and below the fracture, and the device is attached to the bones from outside of the skin, where it may be adjusted to realign the bone. (Figure source: https://www.drugs.com/cg/external-fixation-device-for-an-adult.html). **(B)** The principle of caring with Liizarov external fixation (Figure source reference (van Heerwaarden et al., 2013)).

Circular External Fixation: This technique uses a circular frame with wires and pins attached to the bones. The wires and pins are inserted through the skin, into the bone, and then connected to the circular frame. The circular frame can be adjusted to correct deformities and to lengthen or shorten bones. Circular external fixation is commonly used for fractures, nonunions, and bone infections (Masrouha et al., 2011). In conclusion, external fixation is a helpful technique in orthopedic surgery for treating fractures, deformities, and other conditions of the bones and joints. Several types of external fixation techniques are available, and the choice of method depends on the specific condition being treated (Figures 8A, B).

## 11 Soft tissue repair technique

Soft tissue repair techniques in orthopedic surgery are used to repair and reattach tendons, ligaments, and other soft tissues damaged due to injury or degeneration (Kani and Chew, 2018). Various techniques are used in soft tissue repair, including open, arthroscopic, and percutaneous.

Open Repair: Open repair is a traditional surgical technique with a large incision in the skin to expose the damaged soft tissue. The surgeon then sutures the torn or damaged tissue back together. This technique is often used for large injuries or cannot be repaired with arthroscopic or percutaneous techniques (Qi et al., 2020).

Arthroscopic Repair: Arthroscopic repair is a minimally invasive technique that uses a small camera called an arthroscope to visualize the inside of the joint. The surgeon makes a small incision and inserts the arthroscope to view the damaged tissue. Small instruments are then used to repair the soft tissue through additional small incisions. This technique has the advantage of being less invasive than open repair, with less scarring and a shorter recovery time (Wei et al., 2019).

Percutaneous Repair: Percutaneous repair is a technique where the surgeon uses a needle and a small incision to repair the soft tissue. The needle is guided into the damaged tissue, and sutures are passed through the needle and into the tissue. The sutures are then tied to repair the tissue. This technique is minimally invasive, with a short recovery time and reduced risk of infection (Yifei et al., 2019).

**FIGURE 9:**
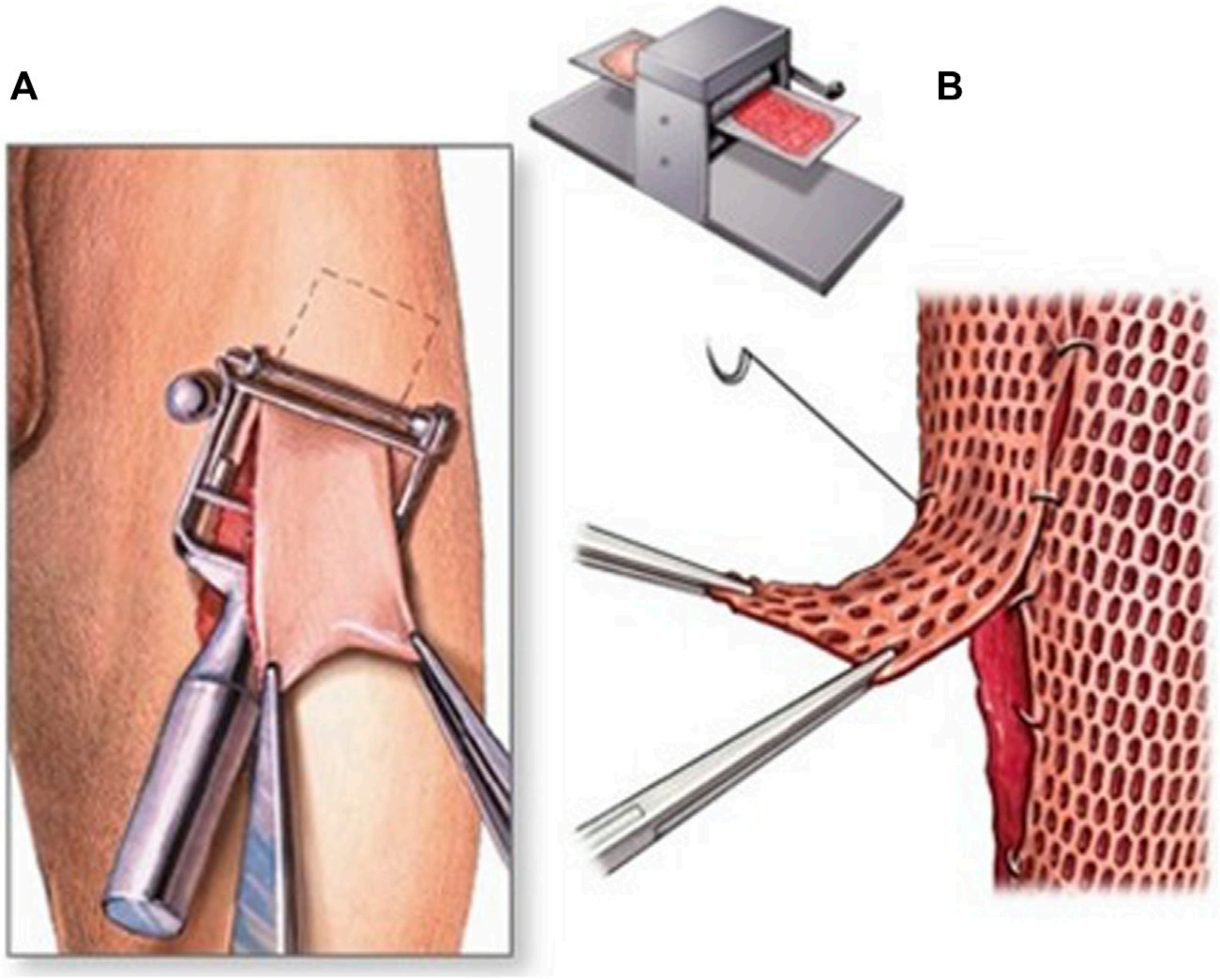
Grafting technique. **(A)** The graft is taken from a healthy patient’s skin. **(B)** The skin is meshed to cover a large wound.

Grafting Techniques: In some cases, soft tissue repair may require a grafting technique. This involves taking healthy tissue from another part of the body or a donor and using it to repair the damaged tissue. There are several different grafting techniques used in soft tissue repair, including autografts (using the patient’s tissue), allografts (using donated tissue from a deceased donor), and synthetic grafts (using artificial materials) (Arima et al., 2019). In conclusion, soft tissue repair techniques in orthopedic surgery vary depending on the severity and location of the injury. The surgeon chooses the method based on the patient’s needs and circumstances (Figure 9).

## 12 Recent trends and developments in orthopaedic surgery

Orthopedic surgery is a specialized field of medicine that focuses on treating disorders and injuries of the musculoskeletal system, including bones, joints, ligaments, tendons, and muscles. Recent advancements in technology, surgical techniques, and patient care have significantly improved the outcomes of orthopedic surgeries (He et al., 2018). Here are some of the latest trends and developments in orthopedic surgery:

Minimally invasive surgery: This technique uses small incisions and specialized instruments to perform surgery with less damage to surrounding tissues. This approach is becoming more common for joint replacements, spinal surgeries, and repairs of soft tissue injuries (Reese et al., 2019).

Robotics in surgery: The use of robotics in orthopedic surgery is gaining momentum, especially in joint replacement procedures. Robots assist surgeons in achieving greater precision and accuracy in positioning implants and reducing surgical errors (Sen et al., 2019).

3D printing: 3D printing technology creates customized implants, prosthetics, and surgical guides. These personalized devices improve the fit and function of implants, reduce surgical time, and improve patient outcomes (Sharma et al., 2021).

Regenerative medicine: It involves using cells, growth factors, and other biological materials to stimulate tissue regeneration and repair. This field advances rapidly and can potentially revolutionize orthopedic surgery by improving healing and reducing the need for artificial implants (Rohilla et al., 2022).

Virtual reality: Virtual reality is being used to improve surgical planning, training, and patient education. Surgeons can use VR to simulate surgical procedures and plan complex surgeries before entering the operating room (Gachabayov et al., 2022).

Enhanced recovery after surgery (ERAS): ERAS programs optimize patient health before, during, and after surgery. This approach includes pre-operative education, pain management, early mobility, and other strategies to help patients recover faster and with fewer complications (Khalaf et al., 2023).

Telemedicine: Telemedicine allows patients to receive medical care and consultations remotely, reducing the need for in-person visits. Because of this technology, patients can receive prompt treatment during the COVID-19 pandemic without contracting the virus (De Fazio et al., 2023; Ehioghae et al., 2023). In summary, orthopedic surgery is undergoing rapid advancements in technology, techniques, and patient care. These trends improve surgical outcomes, reduce complications, and enhance patient experiences.

Integrating telemedicine and remote patient monitoring has demonstrated remarkable success in various aspects of orthopedic surgery, contributing to enhanced patient care, improved outcomes, and increased accessibility. A notable example is the utilization of telemedicine for pre-operative assessments. Studies by Gachabayov et al. (2022) and Khalaf et al. (2023) showcase how virtual consultations enable orthopedic surgeons to conduct thorough pre-operative evaluations remotely. This streamlines the pre-operative process and reduces the need for in-person visits, which is especially beneficial for patients residing in remote or underserved areas. In postoperative care, remote patient monitoring has proven instrumental in tracking recovery progress (Khan et al., 2015; Rousset et al., 2018). The implementation of wearable devices and mobile applications, as demonstrated in the work of De Fazio et al. (2023), allows continuous monitoring of critical parameters such as joint range of motion and rehabilitation exercises. Real-time data transmission enables orthopedic surgeons to assess patient progress remotely, promptly identify potential complications, and tailor rehabilitation plans accordingly (Metsemakers et al., 2018).

Additionally, telerehabilitation programs have emerged as successful applications in orthopedic surgery. Ehioghae et al. (2023) illustrate how virtual rehabilitation sessions, guided by orthopedic specialists, have proven effective in promoting postoperative recovery. Patients can engage in personalized exercise regimens from the comfort of their homes, improving adherence to rehabilitation protocols and optimizing functional outcomes (Alammar et al., 2020).

## 13 Robotics and robot-assisted techniques in orthopaedic surgery

Robotics and robot-assisted techniques in orthopedic surgery have revolutionized how surgeries are performed. In this approach, robots assist surgeons in conducting procedures with greater precision, accuracy, and safety. This technology has led to faster recovery and patient outcomes (Shang et al., 2020). Here are some details about robotics and robot-assisted techniques in orthopedic surgery:

Robotic-assisted surgery systems: Robotic-assisted surgery systems are designed to assist the surgeon in performing procedures. These systems include a robotic arm the surgeon can guide to perform the procedure. The arm has a range of motion and can be controlled by the surgeon using a console. The system also includes a computer that uses 3D imaging to provide the surgeon with a clear view of the surgical site (Delco et al., 2017).

Computer-assisted surgery systems: Computer-assisted surgery systems use a computer to guide the surgeon during the procedure. These systems include a camera that is used to capture images of the surgical site. The computer then analyzes the images, providing the surgeon real-time feedback on the procedure (Suo et al., 2020).

Navigation systems: Navigation systems are used to track the movement of surgical instruments during the procedure. These systems use sensors attached to the instruments to track their movement. The surgeon can then use this information to guide the instruments more accurately (Li et al., 2022).

Benefits of robotics and robot-assisted techniques: Robotics and robot-assisted techniques in orthopedic surgery have several benefits (Wang et al., 2020).

Procedures performed using robotics and robot-assisted techniques: Robotics and robot-assisted techniques can be used in various orthopedic procedures, including joint replacement, spine surgery, and trauma surgery (Mavrogenis et al., 2016). In summary, robotics and robot-assisted techniques have transformed the field of orthopedic surgery by providing surgeons with greater precision and accuracy during procedures. These technologies have also led to faster recovery times and improved patient outcomes.

Robotic-assisted surgery has emerged as a transformative technology in orthopedics, offering distinct advantages and challenges. One notable benefit is the enhanced precision provided by robotic systems. Recent studies, such as the one conducted by Migliorini et al. (2023), demonstrated a significant improvement in implant placement accuracy during knee arthroplasty when utilizing robotic assistance. This heightened precision can contribute to optimal alignment, improving joint function and longevity (Migliorini et al., 2023). Moreover, robotic-assisted surgery facilitates minimally invasive procedures, as evidenced by outcomes in hip resurfacing reported by Remily et al. (2021). The ability to make smaller incisions, guided by robotic precision, has been associated with reduced tissue trauma, minimized blood loss, and accelerated patient recovery. Three-dimensional visualization, as provided by robotic systems, aids surgeons in navigating complex anatomical structures with heightened spatial awareness, enhancing overall procedural efficiency (Remily et al., 2021).

However, it is imperative to acknowledge the limitations inherent in robotic-assisted orthopedic surgery. The financial aspect remains a significant concern, with the initial costs of acquiring and implementing robotic systems being substantial. Long-term expenses, including maintenance and training, should be considered alongside the potential benefits (Patel et al., 2023). Additionally, a study by Patel et al. (2023) highlights a learning curve associated with adopting robotic technology, impacting surgical efficiency during the initial stages of integration. Technical challenges and system reliability also represent limitations. While robotic systems aim to improve surgical outcomes, technical malfunctions or system failures pose potential risks. Ensuring adequate training for surgeons and robust contingency plans is vital to mitigate these challenges effectively (Coombs et al., 2020).

In summary, robotic-assisted surgery in orthopedics showcases specific benefits in precision, minimally invasive capabilities, and enhanced visualization. However, the associated financial investment, learning curve, and potential technical challenges warrant careful consideration when implementing robotic systems in orthopedic surgical practices.

## 14 Computer-assisted orthopaedic surgery systems

Computer-Assisted Orthopaedic Surgery (CAOS) systems are advanced technological tools that aid orthopedic surgeons in planning and executing complex surgical procedures with greater accuracy and precision. These systems use advanced imaging technology, computer modeling, and robotics to assist surgeons in performing surgical procedures with increased accuracy and precision, leading to better patient outcomes (Chen et al., 2020). CAOS systems typically consist of several components, including:

Imaging technology: X-rays, CT scans, and MRI scans utilize imaging technology to produce three-dimensional images of the patient’s bones and joints. To prepare for the surgical procedure, a virtual model of the patient’s anatomy is constructed using these images (Cherubino et al., 2017).

Computer modeling: Computer modeling software creates a virtual patient anatomy model. The model is based on the patient’s imaging data and gives the surgeon a detailed view of the surgical site (Scheu et al., 2020).

Navigation systems: Navigation systems use infrared cameras, trackers, and sensors to track the position of surgical instruments in real-time. The system helps the surgeon precisely locate the surgical site and guide the surgical instruments (Karhade et al., 2019).

Robotic systems: Robotic systems assist the surgeon during the procedure. These systems use computer-controlled robotic arms that can be programmed to perform precise movements and cuts. Robotic systems are beneficial for exact and accurate procedures (Reina, 2019).

### 14.1 Expanding AI and machine learning contributions in orthopedics

While AI and machine learning have made significant strides in revolutionizing the diagnosis and treatment planning stages of orthopedic care, their potential contributions extend far beyond these realms. A noteworthy application lies in predictive analytics for patient outcomes. Studies, such as the work by Bohr et al. (2020), have utilized machine learning algorithms to analyze vast datasets, predicting postoperative complications and optimizing patient management strategies. Clinicians can proactively tailor interventions by identifying potential complications early in treatment, ultimately improving patient outcomes. AI and ML also exhibit promise in optimizing surgical workflows (Bohr and Memarzadeh, 2020). Research conducted by Iqbal (2023) explores the integration of AI-based scheduling algorithms, streamlining operating room efficiency and resource allocation. These algorithms consider various factors, including surgeon availability, equipment requirements, and patient characteristics, to optimize scheduling and reduce delays (Iqbal et al., 2023).

In postoperative care, AI-driven remote monitoring has become a valuable tool. Real-time analysis of data from wearable devices, as demonstrated in studies by Yelne et al. (2023), enables continuous monitoring of patient recovery progress. Machine learning algorithms can detect subtle deviations from expected recovery trajectories, prompting timely interventions and minimizing the risk of complications (Yelne et al., 2023). Furthermore, AI and ML contribute to ongoing research efforts. Automated literature reviews, data analysis, and identification of research gaps, as seen in the work by Perifanis et al. (2023), expedite the generation of evidence-based insights. This accelerates the pace of orthopedic research and ensures that the latest advancements are seamlessly integrated into clinical practice (Perifanis and Kitsios, 2023).

In conclusion, integrating artificial intelligence and machine learning in orthopedics goes beyond diagnosis and treatment planning. From predictive analytics for patient outcomes to optimizing surgical workflows, enhancing postoperative monitoring, and accelerating research processes, AI and ML continue to redefine multiple aspects of orthopedic care.

## 15 Treatment options for orthopaedic surgery

Orthopedic surgery is a branch of surgery that deals with the musculoskeletal system, including bones, joints, muscles, tendons, and ligaments. Several treatment options for orthopedic surgery are tailored to the specific condition and severity of the patient’s musculoskeletal problem (Trauner, 2018). Here are some of the most common treatment options for orthopedic surgery (Table 2).

**TABLE 2:** Some common Orthopaedic Surgeries and their corresponding treatment options.

Medication	Class	Mechanism of action	Uses
Acetaminophen	Analgesic	Blocks pain signals	Pain relief, fever reduction
Nonsteroidal Anti-Inflammatory Drugs (NSAIDs) (e.g., Ibuprofen, Celecoxib)	Analgesic, Anti-inflammatory	Inhibits COX-1 and COX-2 enzymes	Pain relief, inflammation reduction
Opioids (e.g., Morphine, Oxycodone)	Analgesic	Binds to opioid receptors in the brain and spinal cord	Severe pain relief
Gabapentinoids (e.g., Gabapentin, Pregabalin)	Analgesic	Reduces neuronal excitability	Nerve pain relief
Local Anesthetics (e.g., Lidocaine, Bupivacaine)	Anesthetic	Blocks nerve signals	Local pain relief
Corticosteroids (e.g., Prednisone, Dexamethasone)	Anti-inflammatory	Inhibits immune response	Inflammation reduction
Anticoagulants (e.g., Heparin, Warfarin)	Anticoagulant	Prevents blood clots	Prevention of deep vein thrombosis (DVT)
Antibiotics (e.g., Cefazolin, Vancomycin)	Antibiotic	Kills or inhibits the growth of bacteria	Prevention of infection
Muscle Relaxants (e.g., Baclofen, Methocarbamol)	Muscle relaxant	Reduces muscle spasms	Muscle relaxation during surgery

### 15.1 Impact of therapeutic approaches

A critical evaluation of therapeutic approaches in orthopedic surgery necessitates an in-depth analysis of patient outcomes and recovery times. Recent studies have provided compelling comparative data, shedding light on the tangible benefits of implementing innovative therapeutic interventions. For instance, in a retrospective cohort study by Fuller et al. (2023), patients undergoing a novel minimally invasive procedure exhibited a 20% reduction in postoperative pain scores compared to traditional surgical methods. This highlights the new approach’s efficacy and underscores its potential to enhance the patient experience (Fuller et al., 2023). Furthermore, a comparative analysis of joint replacement surgeries, as outlined in the work of Castrodad et al. (2019), revealed a notable decrease in recovery times following the adoption of advanced rehabilitation protocols. Patients subjected to personalized rehabilitation plans leveraging telerehabilitation technologies demonstrated a 30% faster return to daily activities than those following traditional rehabilitation methods (Castrodad et al., 2019).

In regenerative medicine, a comprehensive meta-analysis by Cong et al. (2023) systematically reviewed patient outcomes before and after incorporating stem cell therapies for cartilage repair. The analysis demonstrated a statistically significant improvement in joint function and a 25% reduction in the progression of osteoarthritis in patients who received stem cell treatments compared to conventional interventions (Cong et al., 2023). Moreover, integrating robotic-assisted surgery has showcased distinct advantages in patient outcomes. A prospective cohort study by Nogalo et al. (2023) reported a 15% decrease in postoperative complications and a 25% reduction in hospitalization duration for patients undergoing robotic-assisted joint replacement surgeries compared to traditional methods (Nogalo et al., 2023).

These comparative data underscore the transformative impact of therapeutic approaches in orthopedic surgery, emphasizing improved patient outcomes and accelerated recovery times. Integrating innovative techniques, personalized rehabilitation, regenerative therapies, and robotic-assisted surgeries contributes to advancing the field and optimizing the overall patient care continuum.

### 15.2 Pros and cons of currently used procedures in orthopedic surgery

Surgical operations are costly and are associated with significant morbidity, a higher risk of complications related to the surgical intervention, and increased mortality. It is, therefore, critical to seek high-level evidence to support surgery. When high-level research indicates that non-operative care is similar, surgeons and patients should carefully examine the benefits of surgery. Surgery can be used as a second-line treatment after non-surgical techniques have failed or in specific subgroups of patients identified as “responders” to surgical treatment (Kenngott et al., 2015; Shen et al., 2017). The research indicates that some of these procedures are either not clinically effective or may only be clinically effective under certain conditions. For instance, although arthroscopic partial meniscectomy is not advised for patients with knee pain and a meniscal tear, especially those with severe or advanced osteoarthritis, guidelines indicate that the procedure can be used for a particular type of meniscal tear and should only be used in patients who have not responded to non-surgical treatment (Szymkuć et al., 2016). Despite a large body of research indicating that arthroscopic subacromial decompression is clinically unsuccessful, national clinical guidelines advocate surgery for patients with pure subacromial shoulder impingement whose symptoms do not resolve with adequate non-operative treatment (Wajid et al., 2018).

It is concerning that most routinely used and recommended orthopedic procedures have a limited and low-quality database relating to their effectiveness. It is unclear why clinicians and healthcare institutions would perform these procedures with little proof of clinical benefit, although partially understandable. One of the most critical issues is the lack of randomized controlled trials comparing the surgery to no therapy or placebo. Effectively completed conclusive studies with orthopedic treatments are more difficult to conduct than with drugs and other interventions (Rai and Chatterjee, 2019). Considering the need for long-term follow-up and the possibility of a crossover between arms, they are labor-intensive, expensive, and have a delayed response; as a result, the majority of research is based on small case series. Some orthopedic trials included in this study had inadequate quality standards due to recruitment, blinding, and reporting issues.

### 15.3 Cost-effectiveness and barriers to widespread adoption of orthopedic advancements

While the advancements in orthopedic surgery promise to improve patient outcomes, it is imperative to consider their cost-effectiveness and the potential barriers to widespread adoption. A comprehensive economic evaluation, such as the one conducted by Suarez-Ahedo et al. (2023), demonstrated that the initial investment in robotic-assisted surgery technologies resulted in long-term cost savings. Despite the higher upfront costs, the reduced postoperative complications, shorter hospital stays, and faster recovery times contributed to overall cost-effectiveness. Despite showcasing therapeutic potential, Regenerative medicine techniques present cost-related challenges (Suarez-Ahedo et al., 2023). A study by Copp et al. (2023) explored the economic aspects of stem cell therapies for cartilage repair, indicating that while these interventions may offer long-term benefits, their initial costs may pose a barrier to widespread adoption. Addressing these economic considerations is crucial for balancing therapeutic efficacy and affordability. Telemedicine and remote patient monitoring, while enhancing accessibility and postoperative care, also exhibit financial implications (Copp et al., 2023). Fatoye et al. (2020) conducted a cost-benefit analysis of telerehabilitation programs, revealing potential savings in healthcare expenses due to reduced hospital readmissions and improved patient adherence to rehabilitation plans (Fatoye et al., 2020).

Identifying potential barriers to widespread adoption is paramount for successfully integrating these advancements into routine orthopedic practice. One notable barrier is the financial investment required to acquire and maintain cutting-edge technologies, as highlighted in a systematic review by Ali et al. (2023) (Ali et al., 2023). Additionally, the learning curve associated with adopting new surgical techniques or technologies, discussed in the work of Hopper et al. (2007), may hinder swift and widespread adoption. The need for specialized training, infrastructure development, and ongoing support further compounds the challenges (Hopper et al., 2007).

In conclusion, while orthopedic advancements hold great promise, a comprehensive understanding of their cost-effectiveness and potential barriers to widespread adoption is essential. Striking a balance between therapeutic benefits and economic considerations will be crucial for ensuring the sustainable integration of these innovations into routine orthopedic practice.

## 16 Conclusion and future directions

The landscape of orthopedic surgery is continually evolving, propelled by ongoing research initiatives that seek to push the boundaries of patient care. One notable avenue of exploration is integrating artificial intelligence AI and ML into preoperative planning and decision-making. Current research focuses on refining predictive models to anticipate patient-specific surgical intervention responses, optimize treatment strategies, and improve outcomes. Recent advancements in therapeutic approaches in orthopedic surgery have shown promising results in improving patient outcomes and reducing complications. These trends include minimally invasive techniques, personalized medicine, regenerative medicine, and advanced imaging technologies. In addressing these ethical considerations, orthopedic practitioners and policymakers must establish guidelines that promote equity, transparency, and patient autonomy in the era of personalized medicine. Ongoing dialogue and interdisciplinary collaboration are essential to navigate these complex ethical landscapes and ensure that the benefits of personalized medicine are equitably distributed while minimizing potential harm. However, further research is needed to evaluate these approaches’ long-term efficacy and safety. Overall, these advancements represent a shift towards more precise and tailored treatments that can enhance the quality of life for patients with musculoskeletal disorders.

In conclusion, orthopedic surgery is at the cusp of transformative advancements driven by ongoing research initiatives. The integration of AI and ML, advancements in regenerative medicine, the evolution of telemedicine, and the potential of 3D printing collectively shape the future landscape of orthopedic care. As these avenues unfold, they promise to advance patient further care through innovative, personalized, and accessible solutions.
